# Impact of *Botrytis cinerea* Contamination on the Characteristics and Foamability of Yeast Macromolecules Released during the Alcoholic Fermentation of a Model Grape Juice

**DOI:** 10.3390/molecules25030472

**Published:** 2020-01-22

**Authors:** Richard Marchal, Thomas Salmon, Ramon Gonzalez, Belinda Kemp, Céline Vrigneau, Pascale Williams, Thierry Doco

**Affiliations:** 1 Laboratoire d’Oenologie, Université de Reims Champagne-Ardenne, Moulin de la Housse, BP 1039, 51687 Reims CEDEX 2, France; thomas.salmon@univ-reims.fr; 2 Laboratoire Vignes Biotechnologies et Environnement (LVBE), Université de Haute-Alsace, 33 rue de Herrlisheim, 68008 Colmar CEDEX, France; 3 Instituto de Ciencias de la Vid y del Vino (ICVV - Universidad de La Rioja - Gobierno de La Rioja), Apartado Postal No. 1.042–26080 Logroño, Spain; rgonzalez@icvv.es; 4 Cool Climate Oenology and Viticulture Institute (CCOVI), Brock University, 1812 Sir Isaac Brock Way, St. Catharines, ON L2S 3A1, Canada; bkemp@brocku.ca; 5 Institut Œnologique de Champagne, 9 Rue du Commerce, 51350 Cormontreuil, France; cvrigneau@ioc.eu.com; 6 INRAE, UMR no. 1083, Sciences Pour l’Oenologie, 2 Place Pierre Viala, 34060 Montpellier, France; pascale.williams@inrae.fr (P.W.); thierry.doco@inrae.fr (T.D.)

**Keywords:** *Saccharomyces cerevisiae*, *Botrytis cinerea*, proteins, polysaccharides, foam, proteases, wine, alcoholic fermentation, synthetic grape juice

## Abstract

*Botrytis cinerea* is a fungal pathogen responsible for the decrease in foamability of sparkling wines. The proteolysis of must proteins originating from botrytized grapes is well known, but far less information is available concerning the effect of grape juice contamination by *Botrytis*. The impact from *Botrytis* on the biochemical and physico-chemical characteristics of proteins released from *Saccharomyces* during alcoholic fermentation remains elusive. To address this lack of knowledge, a model grape juice was inoculated with three enological yeasts with or without the *Botrytis* culture supernatant. Size exclusion chromatography coupled to multi-angle light scattering (SEC-MALLS) and sodium dodecyl sulfate-polyacrylamide gel electrophoresis (SDS-PAGE) techniques (AgNO_3_ and periodic acid Schiff staining) was used in the study. When *Botrytis* enzymes were present, a significant degradation of the higher and medium MW molecules released by *Saccharomyces* was observed during alcoholic fermentation whilst the lower MW fraction increased. For the three yeast strains studied, the results clearly showed a strong decrease in the wine foamability when synthetic musts were inoculated with 5% (*v*/*v*) of *Botrytis* culture due to fungus proteases.

## 1. Introduction

Sparkling wine consumption in many cultures is associated with ‘celebrations’ or special occasions, and the visual properties of sparkling wines are of utmost importance for quality [1].

Nowadays, due to this great interest in sparkling wines, sparkling wines are produced in more than thirty countries, with more than 2.5 billion bottles sold in 2015, out of the total world wine production of 36.8 billion bottles (i.e., 6.8% of the world production) [2]. The global sparkling wine market is projected to reach sales volumes of 2963 million bottles by 2021, growing at a compound annual growth rate of more than 2% over the forecast period [3]. Consumers appreciate both a regular and sustained foam ring composed of small and white bubbles on the liquid surface, unceasingly supplied with a continuous effervescence [4,5,6]. 

The duration that sparkling wine foam lasts for is directly related to bubble stability, which itself is dependent on the composition of the liquid film that supports it. The film acts as an elastic barrier formed by proteins, glycoproteins, and polysaccharides, which give the film its viscous-elastic property [7]. Despite their low concentration in sparkling wines, for more than 25 years, studies in Spain and France, and to a lesser/smaller extent in Italy and Portugal have demonstrated the contribution of proteins and polysaccharides (PS) to sparkling wine foam properties [7,8,9,10]. 

These observations were confirmed by studying the enological processes and conditions that reduce wine protein concentration and consequently negatively influence sparkling wine foam. Abdallah et al. [7] showed that ultra-filtered wines deprived of molecules larger than 3.5 and 5 kDa, respectively, did not produce foam, thus confirming that wine foamability is due to the presence of macromolecules. Additionally, the action of bentonite, still largely used as a fining agent for clarification and riddling, and its detrimental effect on foam is well-known [8,9,10,11].

Sparkling base wines contain a large quantity of grape berry glycoproteins [12]. Most of them have an isoelectric point (pI) ranging from 2.5 to 4.5, and molecular weights (MWs) ranging from 12 to 65 kilodaltons (kDa) [13]. Unfortunately, an infection of grape berries with *B. cinerea* leads to a decrease of sparkling wine and base wine foamability [14,15] due to an alteration of the protein composition. The detection of plant proteins in healthy and botrytized Champagne base wines after analysis by sodium dodecyl sulfate-polyacrylamide gel electrophoresis (SDS-PAGE) [16], or 2-dimensional electrophoresis [15] combined with immunoblotting using rabbit anti-must polyclonal antibodies found that some grape berry proteins present in the healthy wine were absent, or degraded, in the contaminated wine. This is probably because some proteins secreted by *B. cinerea* possess proteasic activity [17]. 

In another study dedicated to grape berry proteins isolated from a grape juice, Marchal et al. [18] established a relationship between must protein degradation by fungal proteasic activity and the decrease in the foamability of these plant proteins in a model wine. Nevertheless, no information concerning yeast proteins was provided in these studies.

Aside from proteins and glycoproteins, several studies have identified polysaccharides (PSs) as molecules involved in wine foamability [10]. As for proteins, these wine PSs originate from the grape berry and from the yeast (glucans and mannans or mannoproteins {MPs}). Their composition is largely impacted by the sparkling wine production methods (traditional, transfer, Charmat, carbonation) with consequences for the foamability of the wine [19].

More precisely, the yeast MPs released during alcoholic fermentation and aging on lees are in high MW macromolecules, which are major foam-active compounds/foam stabilizers due to their structure and composition. This favors their adsorption on the gas–liquid interface [7,10,20,21]. It has also been reported that the strains with the greatest autolytic capacity were those releasing the highest quantity of MPs with the highest foamability [22]. In a similar approach using five *Saccharomyces cerevisiae* mutants (obtained by UV mutagenesis) and the parent strain, it was observed that the wine produced with the mutant strain IFI473I exhibited the highest foamability, which was attributed to higher level of MPs released by the strain [20].

Nevertheless, we have very little information concerning the effect of grape juice contamination by *Botrytis* on the biochemical and physico-chemical characteristics of macromolecules released by *Saccharomyces* during the alcoholic fermentation (AF).

## 2. Results and Discussion

### 2.1. Proteins Secreted by Botrytis during the Culture of the Fungus

Proteins released by the fungus *B. cinerea* changed qualitatively and quantitatively during its culture depending on the fungus strain.

**Figure 1:**
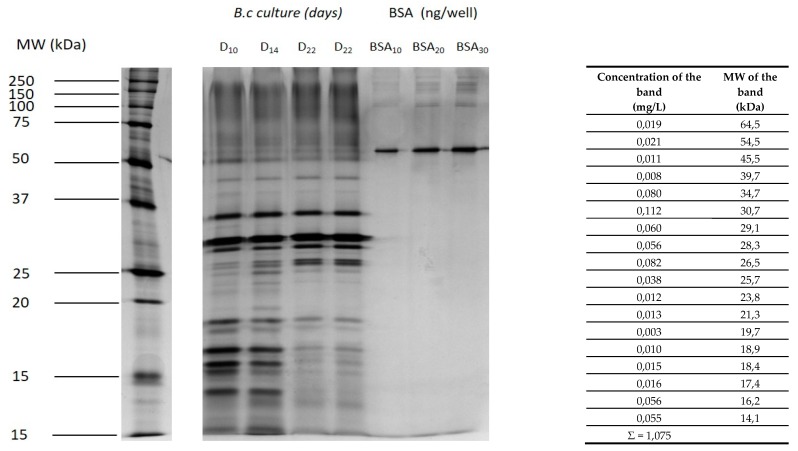
One-dimensional sodium dodecyl sulfate-polyacrylamide gel electrophoresis (SDS-PAGE) analysis of the secreted proteins by *Botrytis cinerea* 630 in a synthetic medium. D_10_, D_14_, D_22_: Days of the culture. The *Botrytis cinerea* culture was filtered, dialyzed (100×), and then concentrated (15×) using Amicon Ultra-4 unit. A total of 15 µL (+5 µL Laemmli buffer) of the ultra-concentrate were loaded/well. The bovine serum albumin (BSA) was loaded at 10, 20, and 30 ng/well. *Botrytis cinerea* protein concentrations for D_22_ were calculated by comparison with the BSA calibration curve.

The *B. cinerea* culture supernatant recovered after 22 days was analyzed in triplicate by SDS-PAGE using bovine serum albumin (BSA) protein for the quantification. In the conditions of the study (no macromolecule or phenolic compounds were present in the Morquer medium), the culture used to contaminate the model juice contained 1.075 mg/L of total proteins and 18 proteic bands clearly identified between 10 and 65 kDa as well as many proteins forming a diffuse stain between 80 and 230 kDa (Figure 1).

The five more intense bands ranged between 28 and 35 kDa (Figure 1). As the culture medium was filtrated after centrifugation, we assumed that these proteins were *Botrytis* extracellular proteins. The SDS-PAGE profile observed in this study was visually quite different from that obtained by Shah et al. [23], probably because the *Botrytis* strains were different. Additionally, the secretome was studied after only five days by Shah et al. [23]. In the present study, we observed strong changes in the secretome proteic composition all along the *Botrytis* culture (Figure 1). For example, the concentrations of the 14.1, 16.2, and 17.4 kDa proteins decreased, and at the opposite end of the scale, the 26.5 kDa protein content increased. This is probably the second main reason for the strong differences between both studies. Finally, the culture medium could also explain some of the differences between both studies. Using LC-MS/MS analysis to provide a qualitative global secretome analysis, Shah et al. [23] identified 113 proteins secreted by *B. cinerea* when the fungus was grown on esterified pectins, but only 89 proteins (−21%) when *Botrytis* was grown on a sucrose liquid medium like ours. These authors also observed that the nature of the proteins (especially the enzymes) was highly dependent on the degree of pectin esterification. Due to these changes in the protein composition, we decided to monitor the proteolytic activity of the secretomes taken between two and 22 days of culture.

### 2.2. Protease Activity on BSA Followed by SDS-PAGE

**Figure 2:**
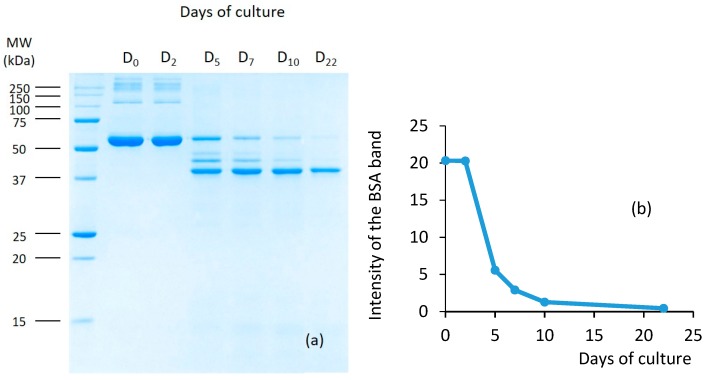
Protease activity on BSA as monitored by SDS-PAGE. (**a**) BSA was incubated for 10 min at 30 °C with the supernatant of *Botrytis cinerea* after 0 to 22 days of culture. Relative MWs (×10^−3^) of the protein standards are given on the left side of the gel. Proteins were stained by CBB. (**b**) Densitometric integration of the native BSA band (the intensities of the bands are expressed by the arbitrary units given by the Bio-Rad integrator).

The proteolysis of the BSA by the *B. cinerea* culture supernatant was detected by SDS-PAGE analysis and Coomassie Brilliant Blue (CBB) staining (Figure 2). The lane D_0_ showed the profile of commercially available BSA without proteolysis (BSA + Morquer medium + glycine-NaOH buffer). The main band corresponded to BSA at around 66 kDa.

This commercial protein also contained higher molecular mass bands in very low quantity, presumably corresponding to the aggregation of BSA molecules. In parallel, a control exclusively containing the *B. cinerea* culture supernatant (without BSA) was also prepared and CBB stained in the same conditions. No signal was observed because of the very low quantity of total protein loaded (3.68 ng/well for *Botrytis* total proteins (i.e., 0.62 ng/well for the more concentrated protein). Therefore, proteins from *B. cinerea* did not interfere with the BSA analysis because the supernatant did not contribute to observable bands at the concentration used in these experiments. In the mixture, the ratio between *B. cinerea* proteins and BSA was 1/2325 (*w*/*w*) (the profile of the *Botrytis* culture alone is shown because no band was observed). Two days after the beginning of the culture, no proteolytic activity was observed (Figure 2, lane D_2_). From D_5_ to D_22_, the intensity of the major band corresponding to BSA continuously decreased with the duration of the *Botrytis* culture, reaching only 2.2% of the initial intensity for D_22_ in only 10 min of incubation. Simultaneously, bands of lower molecular masses appeared to finally disappear with time, thus confirming that the degradation of polypeptides originating from the BSA continued. Waters et al. [24] also showed that BSA was rapidly hydrolyzed in wines by a commercial peptidase preparation containing a range of different peptidases. This preliminary test proved that proteases were effectively present in the *Botrytis* culture inoculated in the model grape juice. 

### 2.3. Botrytis Protease Activity on Yeast Proteins Followed by SDS-PAGE

As for the non-glycosylated BSA, the proteolysis of the yeast proteins released during the AF by the *B. cinerea* culture supernatant was evident in the SDS-PAGE analysis. The model juice containing the *Botrytis* culture supernatant (5% *v*/*v*, corresponding to 53.75 µg/L *Botrytis* total proteins) was analyzed by SDS-PAGE and silver staining. No proteic band was observed. This control experiment clearly demonstrated, as for the CBB staining, that the fungus macromolecules did not give noise to the study of the yeast macromolecules.

**Figure 3:**
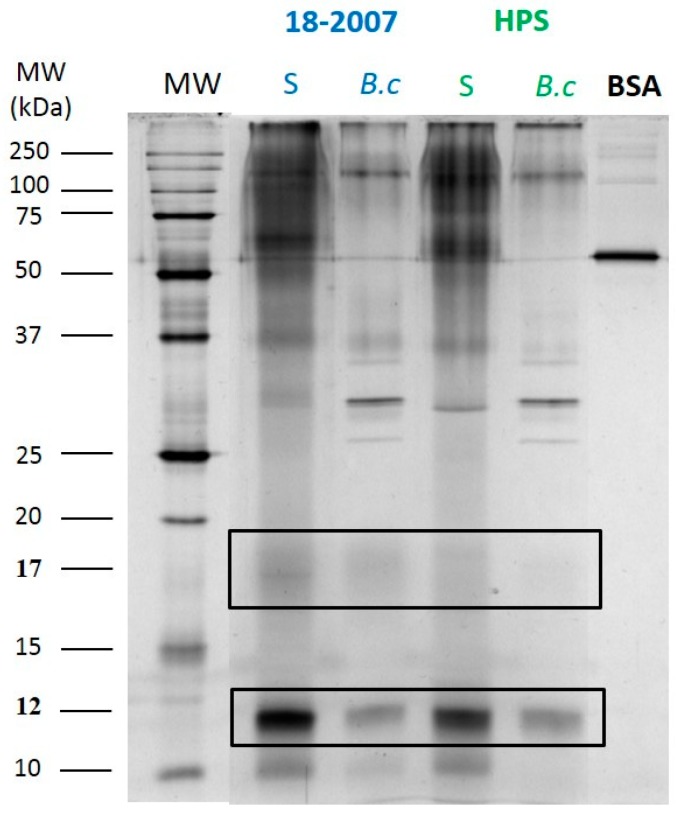
One-dimensional SDS-PAGE analysis of the secreted proteins from the enological *Saccharomyces* strains 18-2007 and HPS. The wines were concentrated 11× for the silver staining. Relative MWs from the standards are given on the left side of the gels. 12 and 17: proteins at 12 and 17 kDa. BSA: 30 ng loaded to calculate the yeast total proteins and the 12 kDa protein in the wines.

In a first step, the wines produced by the 18-2007 and HPS strains were compared by SDS-PAGE + silver staining (Figure 3).

**Figure 4:**
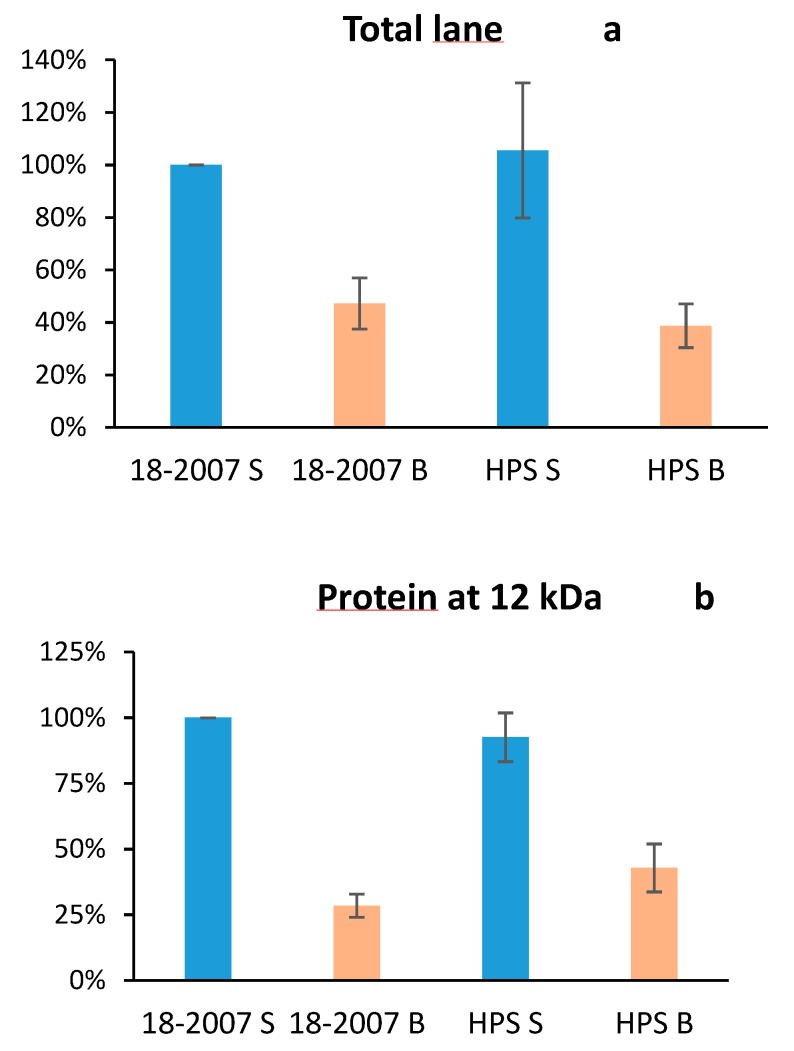
Protein contents in the wines after SDS-PAGE + AgNO_3_ staining and densitometric integration.; (**a**) total proteins; (**b**) protein at 12 kDa.

In the control wines without *B. c* culture, proteic bands forming a stained trail were observed with molecular masses ranging from 45 to 250 kDa as well as proteins at 10.3, 12.1, 16.8/17.6, 30.6, 37.6, 65.3, and 146.5 kDa. Even with the small differences observed, the proteins released by 18-2007 and those released by HPS gave very similar proteic profiles when considering their compositions. Quantitatively, the ANOVA test (Table 1) revealed no significant difference between these two “healthy” wines. The wine 18-2007-H contained 2.66 mg/L total protein (100%) and the wine HPS-H contained 2.90 mg/L total proteins (106%) as determined by silver nitrate staining using the BSA (30 ng loaded in the well) as a reference (Figure 3 and Figure 4a). Quantitatively, the ANOVA test (Table 1) also revealed no significant difference between these two botrytized wines.

These results are in agreement with studies by Martínez-Rodríguez et al. [25], who studied the influence of five yeast strains on the changes of proteins during the sparkling wine production of traditional method wines. With the Bradford colorimetric method, they observed that the concentration of proteins (together with polypeptides higher than 3.5 kDa) was influenced significantly (*P* < 0.05) by the length of time the wines were aged, but not by the yeast strain used during the second fermentation. In fact, the in-bottle fermentation of sparkling wines is currently achieved by only a few commercial *Saccharomyces cerevisiae* strains, and this lack of yeast diversity also leads to a uniformity of the wines’ sensory profiles [26].

With *B. cinerea* culture in the model juice, we detected a strong decrease in intensity for all of the bands for both 18-2007-*B.c* and HPS-*B.c* wines only two months after the beginning of the AF (Figure 3). The intensity of the complete lane 18-2007-*B.c* (1.3 mg/L eq. BSA) decreased by 51.1%, and the lane HPS-*B.c* (0.86 mg/L eq. BSA) decreased by 70.3% when compared to the control wines 18-2007-H and HPS-H, respectively (Figure 3). For these two strains, the presence of *Botrytis* culture in the model juice led to significant decreases (Table 1) of the wine total protein contents. If we consider the band found at 12 kDa (Figure 4b), its intensity in the 18-2007-*B.c* and HPS-*B.c* wines decreased by 72% and 54% when compared to the control wines, 18-2007-H and HPS-H, respectively. 

The comparison of the electrophoretic profiles with and without *B. c* culture (Figure 3) showed a high susceptibility of yeast proteins released during alcoholic fermentation to *B. cinerea* proteases, even if the proteolysis was much lower than that of BSA, which disappeared in 10 min (Figure 2). These different behaviors are linked to the biochemical characteristics of yeast proteins, possibly the presence of glycans capable of partially protecting proteins against proteolytic activities [27].

Nevertheless, the hydrolysis of this yeast’s proteins is highly deleterious because a wine, especially a sparkling wine, is often stored on lees or after corking several years before consumption. It is plausible that the yeast protein degradation continues during wine aging, leading to a wine where *Saccharomyces* proteins will probably completely disappear.

Concerning the nature of these yeast proteins released during fermentation, very little information is available in the literature. The impact of *Botrytis cinerea* grape berry infection on Champagne wine proteins has been studied by Cilindre et al. [28] by using a proteomic approach. Thirty seven spots of interest (differentially and non-differentially expressed in the healthy and botrytized wines) were excised from 2D gels, analyzed by nano-LC-MS/MS, and identified using the NCBInr database. Among these spots, only one in the healthy wine, and one spot in the botrytized wine, were identified as a protein from *S. cerevisiae*, named YJU1 (gi|4814). YJU1 is in fact a *Saccharomyces* cell-wall MP with a theoretical molecular mass of 21.8 kDa and a p*I* of 4.42. The spot was observed in the contaminated wine, but not present in the clean wine. The authors suggested that this protein probably corresponded to that observed in the clean wine before partial modification by fungal proteases. The other thirty five spots originated from the grape berry and corresponded to proteins associated with sugar metabolism (grape vacuolar invertase), proteins involved in plant defenses (thaumatin-like and osmotin-like proteins, chitinases, endochitinases) and proteins secreted by *B. cinerea* (pectinases essentially).

Further experiments that study the exact nature of these *Saccharomyces* proteins impacted by *Botrytis* proteases are necessary. Such studies would provide insight into improving our understanding as to why, and to what extent *Saccharomyces* macromolecules released during the AF are susceptible or resistant to *Botrytis* proteases.

The susceptibility of soluble must protein to *Botrytis* proteases has previously been studied [18] by using a model wine. Using SDS-PAGE, these authors reported that grape berry protein concentration decreased by 35% after only one week, and by 53% after two weeks, whilst the control showed no protein loss. Additionally, it was shown that *Botrytis* proteases were active in the presence of ethanol and SO_2_. In a study conducted with highly botrytized grapes using polyclonal antibodies, the authors found the corresponding botrytized wine lost all the grape juice proteins [16].

In conclusion, when the grape berries are contaminated by *Botrytis* (even partially), the proteases secreted by the fungus during grey mold development are able to destroy most of the macromolecules present in grape juice and wine before, during, and after alcoholic fermentation.

The proteolysis of grape berry proteins by *Botrytis* proteases has been previously reported [16,18]. Waters et al. [24] indicated that grape proteins present in wine produced from healthy grapes show a remarkable resistance to a highly active commercial peptidase preparation, resulting in no significant alteration of the electrophoretic profile by SDS-PAGE. Such discrepancies can be explained by the experimental conditions used in these studies (pH, enzyme concentration, duration of treatment, or temperature) and above all, by the origins of enzymes. These results showed that *Botrytis* proteases are particularly active against the grape juice proteins.

These proteases act as virulence factors allowing the fungus to penetrate/enter the fruit and develop [29]. They successively degrade the flesh of the grape berry, the polypeptides of the grape juice, and finally the proteins of the wine.

Nevertheless, using the SDS-PAGE method and silver nitrate staining (Figure 3), it was not possible to sufficiently detect high MW (glyco) proteins because the glycanic part, which represents generally 71–99% of the molecules, does not react with this staining method. Waters et al. [30] isolated in a red wine a *Saccharomyces* MP protecting white wines from protein haze containing 71.2% of neutral sugars, predominately mannose (97.4%). This MP was only stained with the periodic acid-Schiff’s reagent (no response to the Coomassie Brilliant Blue stain), and was assumed to be an MP due to a decrease in its MW after peptide N-glycosidase F treatment. In another study, Vidal et al. [31] isolated six mannose-rich fractions from a red wine with a very similar composition marked by an overwhelming proportion of mannose (89–97% of the glycosyl residues) and containing no more than 1 to 10% of polyamino acids.

**Figure 5:**
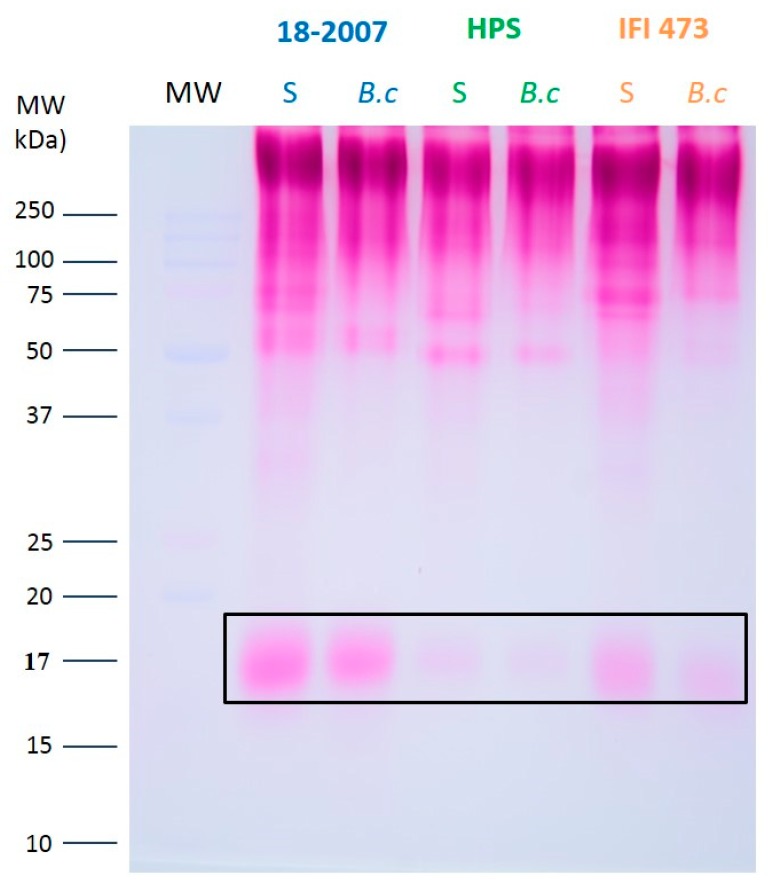
One-dimensional SDS-PAGE analysis of the secreted proteins from the enological *Saccharomyces* strains 18-2007, HPS, and IFI473. The wines were concentrated 45× for the PAS staining. Relative molecular weights (MW) from standards are given on the left side of the gels.

On the basis of these previous studies, we also stained the gels using the periodic acid-Schiff’s (PAS) method [32] after SDS-PAGE for the six wines (Figure 5). 

As for the silver nitrate staining (Figure 3), small differences were observed between the three glycoproteic profiles of healthy wines (Figure 5). These glycoproteins corresponded essentially to yeast macromolecules released during and after alcoholic fermentation [33], and differ from those liberated during the yeast autolysis.

In the control wines (without *B. cinerea* culture), proteins formed a stained trail between 52 and 400–600 kDa as well as proteic bands at 16.8, 31.4, 42, 55.5, 70, and 80 kDa (Figure 5). The very intense zone in the upper part of the gel corresponded to high MW MPs with a SDS-PAGE pattern partly similar to that observed by Waters et al. [30], who noted a 420 kDa MP band at the beginning of the separating gel. These MPs contain N-glycans and mannose chains O-linked to serine and/or threonine, and present a broad stain highlighting the polydispersity of their MWs [30]. This polydispersity was also noted by Vidal et al. [31], who isolated MP fractions ranging from 51 to 527 kDa.

Our results also agree with those of Gonçalves et al. [34], who studied total wine polysaccharides (194 mg/L) isolated from a Vinho Verde wine (Portugal). In their study, MPs represented 32% of the macromolecules with MWs ranging from 53 to 560 kDa, and protein contents ranging from 2.5 to 10.3% of dry matter. 

In Figure 5, we also noted the presence of a diffuse band around 17 kDa (16.5–17.3 kDa) that was visible with the AgNO_3_ staining (Figure 3), thus indicating a high percentage of glycosylation and polydispersity. This protein was much higher in the 18-2007-H wine (100%) than in the HPS-H wine (38%), showing that even if the profiles were similar, the quantity of each protein released during fermentation depends on the enological yeast strain. At the opposite end of the scale, the band silver stained at 12 kDa (Figure 3) did not appear with the PAS staining (Figure 5), probably because of the absence of glycans.

**Figure 6:**
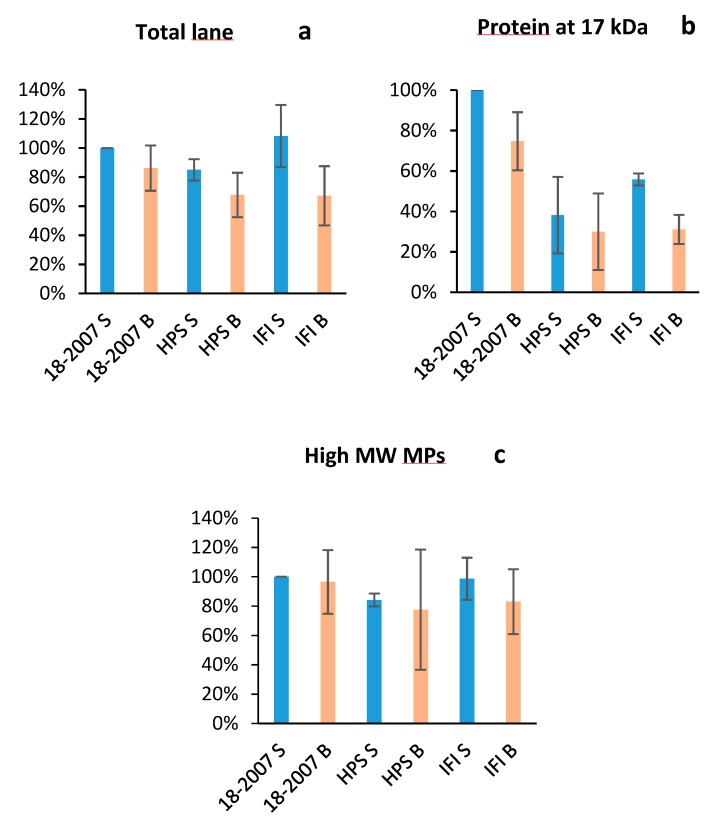
Protein contents in the wines after SDS-PAGE and densitometric integration. PAS staining. (**a**) Total proteins; (**b**) glycoprotein at 17 kDa; (**c**) high molecular weight MPs.

To compare the wine glycoprotein contents (Figure 6a–c), the intensity of the 18-2007-H lane was considered equal to 100% (the strain used in the Champagne region was taken as a reference). The HPS-H and IFI-473-H lanes gave 85 and 108%, respectively. Statistical analysis showed there were no significant differences between the total glycoprotein contents of the clean wines.

The specific PAS staining showed a decrease of the total pink-velvet intensities for the three wines produced from the *Botrytis* contaminated model juice (Figure 5) when compared to the clean wines. These decreases were −14% for 18-2007-*B.c*, −21% for HPS-*B.c*, and −38% for IFI473-*B.c* (Figure 6a). The same can be said for the MPs of high MWs (−4.3% for 18-2007-*B.c*, −9.4% for HPS-*B.c,* and −16.2% for IFI473-*B.c* when compared with clean wines) (Figure 6c), and for the 17 kDa band (−25% for 18-2007-*B.c*, −21.1% for HPS-*B.c*, and −44.7% for IFI473-*B.c* when compared to the clean wines) (Figure 6b).

Two months after the beginning of AF, the high MW molecules seemed to resist the *Botrytis* hydrolytic enzymes more than the glycoproteins with lower MWs. It would be interesting to follow this protein degradation over a longer duration of time to correspond to sparkling wine production and consumption (nine months to five years).

These protein content decreases were due to the presence of *Botrytis* enzymes. We know that *Botrytis* is capable of secreting several kinds of proteases [17]. However, this fungus is also capable of producing other kinds of enzymes such as pectinases [23], laccase, and a multiplicity of plant cell wall degrading glycosidic hydrolases [35]. In our study, the laccase and pectinases had little/no impact on the yeast macromolecules. This was likely due to the model grape juice not containing any pectins or phenolic compounds capable of forming complexes with yeast proteins. *Botrytis* is also able to secrete β-1,3-glucanase as well as β-mannosidase [35] and an enzyme belonging to the family of α-1,6-mannanases [23]. Hence, the decrease of many band intensities (some of them seemed to completely disappear (i.e., proteic bands at 30 and 38 kDa) could also be due to the polysaccharidases/glycanases secreted by the fungus, leading to a partial elimination of N-linked and/or O-linked glycans.

The information provided by the electrophoretic approach with specific and non-specific staining clearly showed the deleterious effect of *Botrytis* enzymatic activities on yeast macromolecules. However, this technique is not precise enough to finely characterize them. For this, we completed the previous approach using size exclusion chromatography-multi-angle laser light scattering (SEC-MALLS) that is able to determine the average MW (M_w_), the radius of gyration (R_g_), the hydrodynamic radius (R_h_), the intrinsic viscosities ([*η*]), and the polydispersities (M_w_/M_n_) of the macromolecules released by the three enological strains during the AF.

### 2.4. Botrytis Enzyme Activities on Yeast Macromolecules Followed by SEC-MALLS

To complete the electrophoretic approach, the proteolysis of the yeast proteins released during the AF by the *B. cinerea* culture supernatant was also found using a SEC-MALLS approach to include all of the macromolecules at the same time. The elution profiles and the MW distributions of the macromolecules provided two principal peaks (datum not shown). The concentration signal was derived from the differential refractometer, whereas the molecular mass was derived from light scattering of the macromolecules released by the three enological strains. The molar mass, the polydispersity index, and the intrinsic viscosity values can be found in Table 2. 

The molar mass appeared to be similar for the macromolecules of the three enological strains without *B. cinerea* culture (around 2.0 × 10^5^ g/mol). Contrary to the clean wines, the wines obtained from the *Botrytis* contaminated model juice had macromolecules with different molar mass centered at 1.4 × 10^5^, 1.7 × 10^5^, and 1.6 × 10^5^ g/mol for 18-2007-*B.c*, HPS-*B.c,* and IFI473-*B.c,* respectively.

The macromolecules released by the three enological strains with or without *B. cinerea* culture were characterized by the same large polydispersity index (*M*_w_/*M*_n_ around 2). The *R*_h_ radius was very low, much lower than 15 nm, and it was not possible to determine the *R*_g_ radius with precision, as it is too small to be measured by SEC-MALLs. The intrinsic viscosity of all the macromolecules was low ([*η*] ± 40 mL/g) regardless of the presence or absence of *B. cinerea* culture during fermentation and the following month. Thus, the decrease in the size of the macromolecules released by the three enological strains in the presence of *B. cinerea* culture did not lead to a decrease in the viscosity of these different macromolecules.

The molar mass distribution analysis of the macromolecule fractions from the *S. cerevisiae* strains 18-2007, HPS, and IFI473, with and without *B. cinerea* culture, are presented in Table 2, and three delimited molar mass ranges were determined (range 1 = 1000‒500,000 g/mol; range 2 = 500,000‒1,000,000 g/mol; range 3 = 1,000,000‒10,000,000 g/mol). The different macromolecule ranges obtained for each healthy strain were compared with those for the same strain with *B. cinerea* culture, and the means compared to one another (ρ-Student test). For the three strains, ranges greater than 500,000 g/mol were significantly reduced (*p* value ≤ 0.05), and halved when fermentation was carried out in the presence of *B. cinerea* culture (Table 2). The consequence of this high MW polysaccharide hydrolysis was that the *B. cinerea* contaminated wines predominantly contained polysaccharides smaller than 500,000 g/mol.

### 2.5. Wine Foam Characteristics

This section presents the impact of *Botrytis* proteases on yeast protein foamability. Using the shaking procedure, we simultaneously followed the foam of the six wines containing only yeast macromolecules to evaluate how *B. cinerea* contamination could influence their foamability.

All of the wines made from model grape juices were between 10.3 and 10.8% alcohol content (*v*/*v*) and between 0.2 and 0.8 g/L residual sugar, except for one IFI473-*B.c* wine (2 g/L). Due to the importance of ethanol on wine foamability, the alcohol contents of the 18 wines were adjusted to the same value (10.8% *v*/*v*) to make the study of the yeast surface active macromolecules with the same surface pressure possible. 

Ethanol concentration influences the wine foamability by decreasing the surface tension of the gas–liquid interface [36], thus impacting the adsorption of other surface-active compounds. A higher ethanol content negatively affects the foamability of sparkling wines. For example, after adding 1.3% ethanol to a base wine to simulate bottle-fermentation, the foamability decreased by 50% [8]. This is the reason why the alcohol content of the wines was adjusted to the same value.

**Figure 7:**
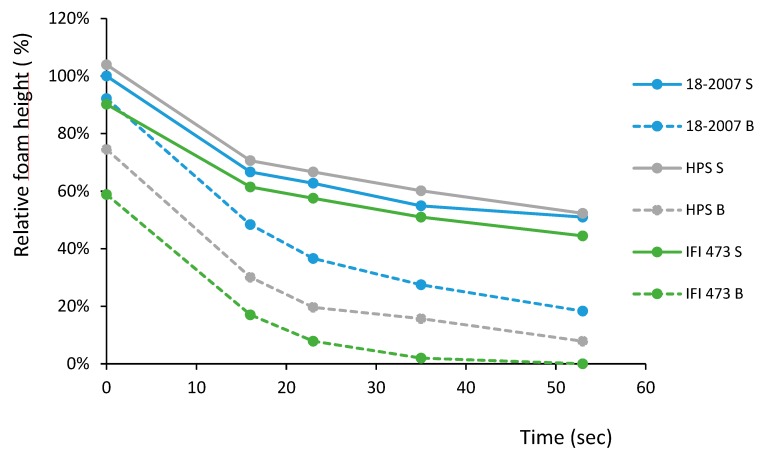
Kinetics of the six wine foam heights with time. S: sound (healthy) wines; B: Botrytized wines. All of the values were calculated through a comparison with the foam height of the sound wine obtained with the 18-2007 strain (18-2007-S = 100% when the liquid and the foam were visually separated after shaking).

In the conditions of our study, the three wines obtained with the three enological strains without *Botrytis* contamination before fermentation presented very similar behavior without a significant difference in foam (at 16, 23, 35, or 53 s) (Figure 7 and Table 3, blue lines). 

This is contrary to a previous study conducted with the parent strain IFI473 and the mutant strains derived from it [37], which found different autolytic capacities and foaming properties [20]. This was attributed to higher levels of proteins and highly glycosylated glycoproteins released by the mutant strain [20]. Thirty-six yeast autophagy-related genes have been identified, with *ATG1*, *ATG17*, and *ATG29* being the main ones along with FPG1, the gene involved in foam formation in *Saccharomyces cerevisiae*. These are the principal genes studied in relation to foaming and autolysis, which is likely to be the topic of further in-depth yeast foaming studies in the future [21].

For the three pairs of wines (clean/botrytized), we found (Table 3) significant differences between the wines. The clean wines presented higher values, and the botrytized wines showed reduced foam (Figure 7), at whatever time considered.

The decrease in foamability was explained by the degradation of the yeast proteins by *Botrytis* proteases. In fact, one can note relationships between the wine total proteins estimated after silver staining and the foam values observed after 16 s (R^2^ = 0.9088), 23 s (R^2^ = 0.9508), and 35 s (R^2^ = 0.9854). 

In a previous study using BSA as a model protein [18], no relationship was observed between the level of degradation of this unique non-glycosylated protein and the decrease of its foamability. This means that protein fragments obtained by partial BSA degradation retained the capacity to produce foam, but at a lower degree than the native BSA. Contrary to previous results obtained with BSA [18], we found strong correlations between foamability and *Saccharomyces* protein content regardless of the status (native or degraded). This discrepancy is not easy to explain because the studied “wines” exclusively contained BSA [18] or was a complex mixture of yeast proteins released during fermentation in the present study. Additionally, the macromolecules released during the AF were mostly highly glycosylated (contrary to BSA) and it is not possible to precisely estimate the role played by the glycans in foam.

After PAS staining, the relationships between total glycoprotein content and foam after 16 s, 23 s, and 35 s (0.65 < R^2^ < 0.67) were lower than those obtained after silver staining (0.91 < R^2^ < 0.98). This was probably because high MW molecules (<400 kDa) appeared less degraded than proteins and glycoproteins lower than 250 kDa, which virtually disappeared. For yeast MPs, whose concentration is around 70–110 mg/L in Champagne base wines [38], the glycosylation level could explain this partial resistance to protease. 

MPs have both hydrophobic domains (proteic moiety) and hydrophilic domains (glycan moiety). Due to this characteristic, they can absorb at the gas/liquid interface and also interact with surface-active materials [21]. The hydrophilic glycans located at the liquid layer are capable of increasing the film viscosity and then delay the drainage of the liquid, thus increasing the bubble lifetime [21]. Proteic fraction of MPs could interact with other proteins to form a more stable film by increasing its viscoelasticity [21]. 

Therefore, MPs are important molecules for wine foamability, but are not able to compensate for the damaging effect of *Botrytis* proteases.

Finally, the strain 18-2007 appeared to better resist the fungus (Table 3, grey lines) when compared to the strains HPS or IFI473 (Figure 5 and Figure 6a–c), resulting in the more affected wine. Nevertheless, the 18-2007-*B.c* foam reached only −51% of the healthy wine foam 35 s after shaking (Figure 7), showing once again that there is no optimal foam when a wine is produced with a *Botrytis* contaminated harvest [14]. These results completely agree with a previous study [18] where the proteins tested were exclusively from grape juice.

The stability of sparkling wine bubbles requires the presence of an adsorption layer at the interface with the gases (air or CO_2_), and wine macromolecules are the main contributors to the formation of these layers. During foam formation, bubbles trap substances such as proteins to stabilize their interfaces [7]. If these components are lacking, then the films are not stable. The surface tension is high and coalescence takes place more easily. Macromolecules in the 5–100 kDa MW range are capable of forming an adsorption layer and monosaccharide analysis of hydrolyzed fractions indicated the presence of mannose, galactose, arabinose, and glucose in decreasing proportions [7,39], thus evidencing that yeast MPs are implicated in the wine foam stability. 

The level of total polysaccharides has also been studied in Spanish wines produced by the traditional method, between three to 26 months after bottling [40]. The highest polysaccharide’ concentrations and improved foamability were detected 18 months after bottling due to the release of polysaccharide compounds from yeast autolysis. Nevertheless, after 18 months, a decrease in foamability was reported, accompanied by an increase in monomeric compounds, likely due to the hydrolytic activity on yeast polysaccharides. This other example clearly indicated that *Saccharomyces* macromolecules released during aging on lees are also sensitive to hydrolysis occurring many months after the death of the yeasts, with negative consequences on wine foam. 

All these studies contribute to strong evidence that MPs are interesting molecules for wine foamability. The addition of yeast proteins could be an interesting alternative to improve sparkling wine foam. Pérez-Magariño et al. [41] reported that such additions did not modify the foam height of sparkling white wines, suggesting that the addition of yeast lees and exogenous proteins at secondary fermentation does not exert an effect on the foaming parameters.

For these reasons, it is necessary to preserve, as much as possible, the potential wine foamability that is strongly affected by many enological conditions. The most damaging, if we accept the use of bentonite, is probably a grape harvest contaminated by *Botrytis*. Even if winemakers have the technical means/skills to produce a sparkling wine, it is necessary to only harvest healthy bunches.

In conclusion, we have shown for the first time that proteases secreted by *B. cinerea* are capable of partially or completely degrading the main macromolecules secreted by three enological yeast strains during and after alcoholic fermentation of a model grape juice. This leads to a strong decrease of sparkling wine foam stability only two months after the beginning of fermentation.

The complete wine making process necessary to produce sparkling wines is between nine months to six years, and even more for top quality sparkling wines. Therefore, it would be very interesting to know whether this *Botrytis* proteolytic activity continues over time. Furthermore, the blend of wines from different vintages could also influence the foam.

## 3. Materials and Methods

### 3.1. Botrytis Cinerea Culture Conditions

The strain used in this study was *B. cinerea* 630, originally isolated from a grape berry sample obtained from one grapevine (field from Boursault Village) in the Champagne area (INRA Versailles, France). It was maintained on tomato-agar plates (food grade tomato juice, Sigma-Aldrich, St. Louis, MO, USA) adjusted to pH 5.5 with NaOH 1 M, containing agar 25 g/L, and sterilized by autoclaving at 121 °C for 20 min. Conidia of 4-week-old cultures were mechanically harvested with a Digralsky spreader in sterilized distilled water and immediately transferred (1.67.10^4^ spores/mL) into a Morquer liquid based medium whose composition was the same as that previously used by Marchal et al. [18]. The conidial suspension was then incubated in a 300 mL Erlenmeyer flask filled with 100 mL at 18 °C on a rotary shaker at 150 rpm for 22 days, with a day–night alternation (12 h with light and 12 h in total darkness). Twenty-two days corresponds to the average time between the mid-veraison and the harvest in the Champagne region. Samples were taken after 0, 2, 5, 7, 10, and 22 days (D_0_ to D_22_), and centrifuged for 10 min at 9500× *g*. The supernatant was then filtered through a 0.45 µm membrane (Alltech, France) and stored at −80 °C before use. The remaining volume (after 22 days) was filtered and directly used to mix with the model juice at 5% (*v*/*v*). 

### 3.2. Isolation and Quantification of B. Cinerea Proteins

The *B. cinerea* supernatants were dialyzed (100×) with distilled water and concentrated (15×) using a low-protein adsorption membrane with a 3000 MW cutoff (Amicon Ultra-4 mL Centrifugal filters, Merck Millipore, Ireland). Ultrafiltration was carried out at 10 °C. The ultraconcentrates (D_0_ to D_22_) were directly analyzed in triplicate by SDS-PAGE (20 µL/well, i.e., 15 µL of 15× *Botrytis* culture + 5 µL of 4× Bio-Rad Laemmli buffer) for protein quantification using the BSA (10–20–30 ng/well) the reference. The model juice containing the *Botrytis* culture supernatant (5% v/v, corresponding to 53.75 µg/L *Botrytis* total proteins) was also analyzed by SDS-PAGE and silver staining.

### 3.3. Evidence of Botrytis Proteasic Activity

The protease activity of the *B. cinerea* culture supernatant was assayed by following BSA degradation using the SDS-PAGE and CBB staining method. The 8.3 cm × 7.3 cm dimension and 0.75-mm-thick slab gel was composed of a 5% polyacrylamide (T = 5% and C = 2.7%) (Bio-Rad Laboratories, Inc., Beijing, China) stacking gel and 13.5% polyacrylamide (T = 13.5% and C = 2.7%) separating gel. A vertical Mini-PROTEAN® III electrophoresis apparatus (Bio-Rad Laboratories S.r.l., Segrate, Italy, and Bio-Rad Laboratories Singapore Pte. Ltd.) was used to run the gel at a constant voltage of 150 V until the bromophenol blue tracker dye reached the gel bottom (usually 65 min at room temperature). The maximum sample volume that can be loaded per well is 33 µL (10 well combs). A total of 100 µL of BSA dissolved in distilled water at 10 g/L was mixed with 400 µL of *B. cinerea* culture (after 0, 2, 5, 7, 10, and 22 days of culture) + 1500 µL of citrate-phosphate buffer (0.1 M pH = 3.5). The mixture was incubated for 10 min at 30 °C. *Botrytis* proteolytic activities were then blocked by adding 100 µL of glycine-NaOH buffer (0.1 M pH = 10). Samples containing BSA + *B. cinerea* culture (3 vol.) were then diluted with the 4x Bio-Rad Laemmli buffer (1 vol.) and 24 µL were loaded into the wells for each analysis (without β-mercaptoethanol nor boiling treatment). After SDS-PAGE + CBB staining [18], BSA proteolysis was followed by monitoring the decrease in absorbance of the BSA band at around 66 kDa (Bio-Rad Doc XR^+^ scanner). Each value obtained by densitometry integration corresponded to the average of three measurements. 

### 3.4. Model Juice Alcoholic Fermentation

The three enological yeast strains of *Saccharomyces* used in this study were 18-2007 (Institut Oenologique de Champagne, Epernay, France), HPS (Lallemand, Toulouse, France), and IFI473 (Institut Oenologique de Champagne, Epernay, France). These yeast strains are used for the AF in tanks as well as the in-bottle fermentations to produce sparkling wine by the traditional method. Yeasts (1 g) were rehydrated over 20 min at 32 °C in a solution (20 mL) containing model juice and water (v/v). After rehydration, the yeast suspension was inoculated (0.9 mL, corresponding to 0.2 g/L of dry yeasts) to the buffered model juice (212.85 mL). Composition of the juice buffer per liter was as follows: KH_2_PO_4_, 935 mg; NH_4_H_2_PO_4_, 561 mg; (NH_4_)_2_ SO_4_, 187 mg; MgSO_4_, 467 mg; NaCl, 94 mg; CaCl_2_, 94 mg; biotin, 187 µg; inositol, 1.87 mg; pyridoxal, 1.87 mg; pantothenate Ca, 1.87 mg; thiamine chlorhydrate, 1.87 mg; nicotinic acid, 0.468 mg; H_3_BO_3_, 0.47 mg; KI, 0.094 mg; FeCl_3_, 0.752 mg; Zn SO_4_, 0.188 mg; CuSO_4_, 0.0376 mg; MnSO_4_, 0.376 mg; (NH_4_)6Mo_7_O_24_, 0.188 mg; D-glucose, 187.5 g; citric acid, 0.5 g; tartaric acid, 3 g; malic acid, 6 g; Tween 80, 13.5 mg; and ergosterol, 0.3 mg, in distilled water. After solubilization of all the compounds, the model juice was filtered through a Durapore 0.45 µm PVDF membrane (Merck Millipore, Carrigtwohill, County Cork Ireland) before yeast inoculation.

The alcoholic fermentations were carried out in triplicate in 250 mL sterile Erlenmeyers filled with 213.75 mL of inoculated model juice. An addition of 5% (v/v) *Botrytis* culture supernatant (11.25 mL) in the model juice corresponded to the “*Botrytis* contaminated” must. The model juice (+5% *v/v* Morquer medium) without *Botrytis* culture supernatant corresponded to a synthetic “healthy” must. The Erlenmeyers were closed with cotton. The AF were followed by weighing the Erlenmeyers. When the weight was unchanged for 48 h, 7 g/L SO_2_ was added to the wine. Then, the residual sugars and alcohol contents were determined. The wines produced from the model must are noted as follows: Yeast strain-H for the wine obtained from healthy model juice and Yeast strain-*B.c* for the wine obtained from the *B. cinerea* contaminated model juice.

### 3.5. Yeast Protein Analysis by SDS-PAGE

Discontinuous SDS-PAGE was performed according to the method of Laemmli [42] using slab gels (dimension 8.3 × 7.3 cm, 0.75 mm thick) with the same conditions and the same equipment as those in Section 2.3. The wines were dialyzed (100×) against distilled water (3 kDa Amicon Ultra-4 mL Centrifugal filters, Merck Millipore, Ireland) and then concentrated 11× for the silver staining or 45× for the periodic acid Schiff (PAS) staining. Protein samples (3 vol) were diluted with the 4x Bio-Rad Laemmli buffer (1 vol) and 24 µL were loaded into the wells for each analysis without boiling treatment.

Five-fold diluted standard 10–250 kDa proteins (Precision Plus Protein ^TM^ Unstained Standards, Bio-Rad, USA) were used as MW markers and 1 µL was loaded when proteins were silver stained. No-diluted pink and blue pre-stained standard proteins from 10 to 250 kDa (Precision Plus Protein ^TM^ Dual Xtra Standards, Bio-Rad, USA) were used as MW markers and 5 µL was loaded when proteins were stained according to a PAS method. The MWs of unknown proteins were calculated from the linear regression equation of log MW versus mobility. After migration, gels were silver-stained according to the protocol described by Rabilloud et al. [43] or PAS stained [32] to characterize the presence of sugars or stained with Coomassie Brilliant Blue [18] for *Botrytis* proteasic activity evidence. For each sample, the gels were carried out in triplicate. After coloration, the SDS-PAGE gels were scanned with a Bio-Rad Doc XR^+^ scanner and analyzed for the determination of the MW and the quantification using the Image Lab software. The BSA was regarded as a standard for each protein band silver stained and their contents were expressed as mg/L eq. BSA.

### 3.6. Isolation of Polysaccharide Fractions

The complex carbohydrates were precipitated after ethanolic dehydration. This was performed by adding ethanol (95% acidified by HCl 0.5%) to obtain a final concentration of 80% ethanol [44,45]. After overnight at 4 °C, the precipitates (total wine polysaccharides) were recovered by centrifugation (30 min, 9954× *g*, 4 °C), dissolved in water, and then freeze-dried. Then, the supernatant, which contained the total oligosaccharide fraction, was recovered, and dialyzed extensively against distilled water (MWCO 1 kDa), concentrated, and freeze-dried. 

### 3.7. Multi-Detector High Performance Size Exclusion Chromatography (HPSEC)

HPSEC experiments were performed using a Shimadzu HPLC system (Shimadzu, Kyoto, Japan) coupled to four detectors: multi-angle light scattering (MALLS) operating at eighteen angles from 10 to 160° (Dawn Heleos II, Wyatt, CA, USA), differential refractometer (Optilab T-rEX, Wyatt, CA, USA), on-line viscometer (VISCOSTAR II, Wyatt, CA, USA), and UV–Vis detector activated for a 280 nm wavelength (SPD-20A, Shimadzu, Japan). The system was composed of a set of three columns: a pre-column Shodex OHPAK SB-G, followed by two columns in series, Shodex OHPAK SB 804 HQ and OHPAK SB 805 HQ. Polysaccharide fraction in solutions at 1 g/L concentration were eluted through the system at 30 °C with 0.1 mol/L solution of LiNO_3_ at a constant flow rate of 1 mL/min. Data were analyzed using ASTRA software (Wyatt Technologies, Santa Barbara, CA). Average MWs, radius of gyration (Rg), hydrodynamic radius (Rh), intrinsic viscosity ([η]), and polydispersity (Mw/Mn) were calculated using a refractive index increment (dn/dc) of 0.145 mL/g [46].

### 3.8. Sugar and Ethanol Contents

The analytical methods recommended by the Compendium of International Methods of Wine and Must Analysis [47] were used to determine the sugar (g/L) and the alcohol (% *v*/*v*) contents. An Anton Paar DMA 48 Density Meter (Anton Paar, Courtaboeuf, France) was used to analyze the sugar content, which was calculated according to the mass per volume unit. The alcohol content was determined by near-infrared spectrometry with a SpectraAlyzer (Zeutec GmbH, Rendsburg, Germany). The alcohol content of the 18 wines (6 trials × 3 bottles/trial) obtained after AF were adjusted to 10.8% *v/v* (using 99.9% *v/v* ethanol). The dilution induced by ethanol adjustment was between 0 and 0.5% (*v*/*v*) and considered as negligible.

### 3.9. Foamability

The foamability of the six wines produced from a model must by the three different yeast strains, with and without *Botrytis* culture, were measured in triplicate by a standardized shaking process. Glass tubes (18 cm in height, internal diameter 1.36 cm) were filled with 18 mL of wine representing 11 cm in height in the tube. 

The automated shaker enabled six wines to be studied simultaneously, thus reducing the experimental error that can occur with a sparging procedure such as the Mosalux system used in many studies dedicated to sparkling base wines [8,9,48]. The rack with the six test tubes (containing the three healthy wines and the three *Botrytis* contaminated wines) were attached to a mechanical arm. The arm made a return rotation of 90 degrees in 1 s (a round trip per second). Therefore, the movement was exactly the same for the six tubes. Ten movements were applied to generate the foam by shaking.

After agitation, pictures of the tubes (in vertical position) were taken to follow the decrease of the foam. Before the pictures, a ruler (graduation = 1 mm) was placed between two of the tubes to have a scale. The pictures were taken during 53 s (i.e., when the foam of one of the tubes completely disappeared (0 mm). The foam height was then measured on the pictures when it was possible to visually detect a clear separation between the foam and the wine (t_0_), even if small bubbles were still ascending in the wine. Results were obtained in mm. The foam height was not determined by a sensor, but visually because the foam is a sensory (visual) characteristic of the wine.

The value obtained at t_0_ for the clean wine, produced by the strain 18-2007 without *Botrytis* (18-2007-H), was considered to be equal to 100%. All other measures were calculated in a percentage proportional to this value. Camera specifications and pictures characteristics were as follows: Nikon D800, lens AF-S NIKKOR 24–85 mm, 1:3, 5-4, 5G, iso 640, F/4.5, time 1/100 s, focal length 85 mm.

### 3.10. Statistics

The standard deviation and ANOVA were carried out using XLSTAT software.

## Figures and Tables

**Table 1:** Total protein contents analyzed by ANOVA. S***, Significant statistical differences (*P* < 0.001) between clean and botrytized wines (yellow). NS, Non-Significant statistical differences (*P* > 0.05) between clean wines (blue) and between botrytized wines (grey). F_exp_, experimental Fisher value. F*, critical Fisher value.

		F_exp_	F*
18-2007 S vs. 18-2007 B	S***	227.02	25.41 (*P* *<* 0.001)
HPS S vs. HPS B	S***	46.95	25.41 (*P* *<* 0.001)
18-2007 S vs. HPS S	NS	0.36	5.32 (*P* *<* 0.05)
18-2007 B vs. HPS B	NS	3.38	5.32 (*P* *<* 0.05)

**Table 2:** Rheological parameters ^a^ and distribution analysis ^b^ determined by light scattering obtained for the macromolecules released by the three enological *S. cerevisiae (S.c.)* strains 18-2007, HPS, and IFI473, with or without *B. cinerea* culture.

	*S. c.* 18-2007		*S. c.* 18-2007 + *B. Cinerea*			*S. c.* HPS		*S. c.* HPS + *B. Cinerea*			*S. c.* IFI 473		*S. c.* IFI 473 + *B. Cinerea*		
	Means	*S.D*	Means	*S.D*	*p*-Value	Means	*S.D*	Means	*S.D*	*p*-Value	Means	*S.D*	Means	*S.D*	*p*-Value
**M_n_ (g/mol)**	207 833	*±27 775*	143 850	*±8 980*	**0.057**	207 350	*±6 250*	170 433	*±23 481*	**0.058**	214 466	*±15 315*	161 100	*±12 162*	**0.026**
**M**_**w**_** (g/mol)**	423 033	*±52 465*	313 050	*±37 547*	**0.086**	361 550	*±129 40*	24 666	*±24 808*	**0.158**	478 766	*±41 101*	412 050	*±23 263*	**0.136**
**M**_**w**_**/M**_**n**_	2.03	*±0.07*	2.17	*±0.12*	**0.232**	1.74	*±0.00*	1.91	*±0.12*	**0.174**	2.23	*±0.10*	2.55	*±0.04*	**0.029**
**R**_**z**_	19.83	*±1.81*	24.15	*±6.43*	**0.322**	17.44	*±0.50*	16.76	*±0.65*	**0.311**	23.03	*±0.90*	24.75	*±1.48*	**0.194**
**[*****ŋ*****] (mL/g)**	40.71	*±0.62*	35.16	*±3.91*	**0.078**	38.68	*±0.18*	43.27	*±1.12*	**0.012**	45.22	*±2.85*	42.58	*±2.92*	**0.389**
**R**_**h**_****	12.93	*±0.52*	11.225	*±0.75*	**0.054**	12.26	*±0.16*	12.24	*±0.20*	**0.910**	3.57	*±0.39*	12.52	*±0.52*	**0.078**
**mass (µg)**^**c**^	51.4	*±10.1*	56.7	*±4.8*	**0.455**	47.6	*±1.02*	38.1	*±4.3*	**0.062**	50.8	*±0.4*	43.7	*±3.0*	**0.052**
**Range 1**	77.2	*±2.8*	83.2	*±4.0*	**0.099**	80.1	*±5.3*	89.4	*±5.2*	**0.098**	80.8	*±1.5*	87.5	*±1.9*	**0.009**
**Range 2**	18.2	*±1.8*	13.7	*±2.4*	**0.066**	14.3	*±4.1*	7.9	*±4.9*	**0.158**	14.7	*±1.0*	9.9	*±1.0*	**0.004**
**Range 3**	4.6	*±1.4*	2.8	*±1.3*	**0.188**	5.0	*±1.2*	2.4	*±0.6*	**0.031**	4.16	*±0.9*	2.2	*±0.6*	**0.042**

^a^ Molar-mass distributions, *Mw*, *Mn*, polydispersity index (*M*_w_/*M*_n_); *R*_h_ radius determined by coupling size exclusion chromatography performed on two serial Shodex OH-pack columns with a multi-angle light scattering device (MALLS); -MALLS in 0.1 M LiNO3 (*dn/dc* = 0.146 mL/g). Intrinsic viscosity ([*η*]) determined by a differential viscometry detector equipped with a four-capillary bridge design. ^b^ Molar mass range determined by SEC-MALLS: range 1 = 1000‒500,000 g/mol; range 2 = 500,000‒1,000,000 g/mol; range 3 = 1,000,000‒10,000,000 g/mol; ^c^ Correspond to the mass of each injected fractions calculated by SEC-MALLS.

**Table 3:** ANOVA of the different wines’ foaming properties between 16 and 53 s. S, Significant statistical differences for *P* < 0.05. S *, Significant statistical differences for *P* < 0.025. S **, Significant statistical differences for *P* < 0.01. S ***, Significant statistical differences for *P* < 0.001. NS, Non-Significant statistical differences (*P* > 0.05). Comparisons between sound S (healthy) and botrytized B wines (yellow), between sound wines (blue) and between botrytized wines (grey).

Time (s)	T16	T23	T35	T53
18-2007 S vs. 18-2007 B	S *	S *	S	S
HPS S vs. HPS B	S *	S	S	S *
IFI S vs. IFI B	S ***	S ***	S ***	S ***
18-2007 S vs. HPS S	NS	NS	NS	NS
18-2007 S vs. IFI S	NS	NS	NS	NS
HPS S vs. IFI S	NS	NS	NS	NS
18-2007 B vs. HPS B	NS	NS	NS	NS
18-2007 B vs. IFI B	S*	S	S	S
HPS B vs. IFI B	NS	NS	NS	NS
